# Symptom dimensions in people affected by long-term neurological conditions: a factor analysis of a patient-centred palliative care outcome symptom scale

**DOI:** 10.1038/s41598-019-41370-3

**Published:** 2019-03-21

**Authors:** Rebecca Wilson, Nilay Hepgul, Romi A. Saha, Irene J. Higginson, Wei Gao

**Affiliations:** 1 Cicely Saunders Institute of Palliative Care, Policy & Rehabilitation, Florence Nightingale Faculty of Nursing, Midwifery & Palliative Care, King’s College London, Bessemer Road, London, SE5 9PJ UK; 2 Hurstwood Park Neurological Centre, Brighton and Sussex University Hospitals, Lewes Road, Haywards Heath, RH16 4EX UK

## Abstract

Long-term neurological conditions (LTNCs) often cause debilitating symptoms. Better understanding of symptom dimensions in LTNCs is needed to support health professionals and improve care. This can be achieved by exploring the factor structure of a standardised measure of symptoms in LTNC patients. The symptom subscale of the Integrated Palliative Outcome Scale for LTNCs (IPOS Neuro-S24) comprises 24 items measuring symptom severity. Descriptive statistics and psychometric properties of the scale were assessed, followed by differential item functioning (DIF), exploratory factor analysis (EFA) and confirmatory factor analysis (CFA). Data from N = 238 patients were analysed. The mean IPOS Neuro S-24 score was 27.0 (possible range 0–96) and floor effects were found for 21 items. The scale had good internal consistency (Cronbach’s alpha = 0.77). Weak evidence of DIF was found for nine items. All but one item (falls) loaded onto four factors with loadings > 0.3. The factors represented four clinically meaningful symptom dimensions: fatigue, motor symptoms, oral problems and non-motor symptoms. We identified a reliable four-factor structure of symptom experience in LTNC patients. The results suggest that symptom dimensions are common across LTNCs. The IPOS Neuro S-24 is an appropriate tool to measure symptoms in LTNC patients, which may improve care.

## Introduction

Long-term neurological conditions (LTNCs), including multiple sclerosis (MS), idiopathic Parkinson’s disease (IPD), multiple system atrophy (MSA), progressive supranuclear palsy (PSP) and motor neurone disease (MND), are non-communicable, progressive diseases that can affect patients’ physical capability, sensory function, cognition, behaviour, communication and emotions^1^.

Research has previously underestimated the burden of LTNCs by considering only mortality and not disability related outcomes^2^, thus further research into the symptom experience of LTNC patients is warranted. Symptoms in LTNCs are common, multiple and insufficiently studied in the more advanced stages of disease, yet such information is vital to improve care^3–5^.

Investigating patient symptomatology requires the use of a valid measurement tool. Exploring a measure’s psychometric properties often includes identifying the tool’s internal consistency (a measure of scale reliability) and any floor and ceiling effects (where respondents excessively score at the lowest or highest end of the scale, respectively). The psychometric development of a validated tool may also include factor analysis, a statistical approach to identifying the internal structure of a measure by exploring the relatedness of individual items.

Factor analysis has been used previously to assess symptom profiles for cancer^6,7^, heart failure, kidney disease^8^ and acute mania^9^. Assessing the factor structure of symptoms may contribute to the development of evidence-based diagnostic tools^9^. This could benefit both health professionals and patients, as care can be focused on managing groups of symptoms, rather than inefficiently treating single symptoms^10^.

A previous factor analysis of eight symptoms in LTNC patients^11^ aimed to validate a shortened palliative care outcome measure for this patient group, but did not explore the factor structure of the whole spectrum of symptoms and identify clinically meaningful symptom clusters. No other studies have explored the factor structure of symptoms across LTNCs.

The present study aimed to explore the factor structure of a 24 item measure of symptoms in LTNC patients. It was hypothesised that, including a wider variety of symptoms, a factor analysis would identify a multi-factorial internal structure, which would illustrate distinct symptom dimensions across LTNCs.

## Methods

### Sample

Patients were recruited for a trial investigating the effectiveness of a palliative care intervention from seven sites across the United Kingdom between April 2015 and August 2017 (the OPTCARE Neuro trial, NIHR trial registration number: ISRCTN18337380). Eligibility criteria were that patients had a diagnosis of either MS, Parkinsonism or related disorders (including IPD, PSP and MSA), or MND. Patients were also required not to have received specialist palliative care in the last six months, to have an unresolved symptom not responding to usual care and have at least one of the following: another unresolved symptom, cognitive problems or complex psychological/social needs. Patients were referred to the study by their neurology teams. The sample for the present study was restricted to patients with complete symptom data at baseline.

### Ethical approval and procedure

Ethical approval was obtained from the National Research Ethics Service Committee London South East (REC number: 14/LO/1765). All methods were performed in accordance with the relevant guidelines and regulations. After referral to the OPTCARE Neuro trial and the provision of a patient information leaflet, research nurses visited patients in their homes to obtain written informed consent and collect baseline data, using the Integrated Palliative Outcome Scale Neuro S-24 (IPOS Neuro S-24). Baseline data from the OPTCARE Neuro trial were used for this analysis.

### Measures

#### IPOS Neuro S-24

The family of Palliative Outcome Scales (POS) measures the palliative care needs of patients^12^. The IPOS Neuro-S24 was developed to assess symptoms in LTNC patients. It includes 24 symptom specific items, scored on a 5-point Likert scale from 0 (not at all) to 4 (overwhelmingly), where patients responded how they had been affected by that symptom in the last 3 days^4^. Symptoms included in the scale had previously been measured in MS, IPD, PSP and MSA patients^13^ and eight core symptoms showed promising psychometric properties following validation in MS, IPD, MSA and PSP patients (forming the IPOS Neuro-S8)^11^.

### Data analysis

Descriptive analyses were used to describe item specific scores, using the mean and standard deviation. Frequency distributions for item-level responses scoring 0 (no problem) to 4 (overwhelmingly) were expressed as frequency and percentage. Floor and ceiling effects were measured by the number of respondents receiving the minimum (0) and maximum (4) score on the individual items. Floor and ceiling effects were deemed present when more than 15% of patients reported the extreme scores^11^. The internal consistency (an indicator of reliability) of the measure was calculated using Cronbach’s alpha coefficient. Differential Item Functioning (DIF) was used to identify differences (in individual item scores) between MS and IPD patients.

Exploratory factor analysis (EFA) was used to initially evaluate dimensions of the measure. As items were ordered categorical, polychoric correlation was used. EFA using principal factors extraction with oblique rotation was performed. A scree plot and minimum Eigen value of 1.0 were used to identify the number of factors to retain; a minimum factor loading of 0.3 was also specified.

The EFA output was used to inform the confirmatory factor analysis (CFA). The CFA model fit was assessed using fit indices (RMSEA, SRMR, CFI and TLI). The following thresholds were used to indicate a good fitting model: RMSEA < 0.05, CFI > 0.95, TLI > 0.95 and SRMR < 0.08^14,15^. The modification indices command (*estat mindices*) was used to identify any further factor loadings or covarying error terms that would improve model fit; these were then added to the model. Correlation coefficients were used to assess correlation of factors. Parameters described by Evans *et al*.^16^ were used to classify weak (<0.4), moderate (0.4–0.59) and strong (>0.6) correlations. Stata 14 was used to perform data analysis.

## Results

### Sample characteristics

Data were collected from 350 patients; 284 had complete data for all 24 symptoms. Patients with incomplete symptom data (N = 66) were excluded. Patients lacking mental capacity (N = 39) were missing data as their data were collected via a carer and only patient-reported data were used in analysis, thus these data were missing at random (MAR). The remaining 17 patients with incomplete symptom data were missing data completely at random (MCAR). A further one patient was excluded as their diagnosis (‘other’) did not meet inclusion criteria. The analytical sample was 283. The descriptive statistics for the 67 excluded patients were comparable to the analytical sample: mean age = 67 years, 51% female, median years since diagnosis = 13, the majority were married, living with spouse, primary/secondary school educated and of white ethnicity. The characteristics of the analytical sample are presented in Table 1.

**Table 1:** Sample characteristics (N = 283).

Age	Mean (SD)	66.6 (11.5)
Gender	% Woman	48.4%
Diagnosis, N (%)	Multiple sclerosis	119 (42.1)
	Idiopathic Parkinson’s disease	113 (39.9)
	Multiple system atrophy	9 (3.2)
	Progressive supranuclear palsy	20 (7.1)
	Motor neurone disease	22 (7.8)
Duration of illness since diagnosis up to enrolment (in years)	Mean (SD)	12.9 (10.8)
	*Missing, N*	1
Marital status, N (%)	Single	27 (9.5)
	Widowed	32 (11.3)
	Married/civil partner	188 (66.4)
	Divorced/separated	35 (12.4)
	*Missing, N*	1
Living arrangement, N (%)	Alone	54 (19.1)
	With children	19 (6.7)
	With spouse/partner	146 (51.6)
	With spouse/partner and children	35 (12.4)
	With friend(s)	3 (1.1)
	Other	26 (9.2)
Educational attainment	Primary/lower secondary school	106 (37.6)
	Upper secondary school/vocational training	100 (35.5)
	Tertiary	76 (27.0)
	*Missing, N*	1
Ethnicity	White	263 (92.9)
	Black/African/Caribbean/black British	6 (2.1)
	Other ethnic group	13 (4.6)
	Prefer not to say	1 (0.4)

### Descriptive statistics and psychometric properties

The mean total score of the IPOS Neuro-S24 was 27.0 (SD = 10.3; Range 3–59) (possible range: 0–96). Kurtosis was 3.2 and skewness was 0.5, indicating near normal distribution, which was confirmed by visual inspection of a histogram. The highest scoring items were poor mobility, fatigue and problems using legs (see Table 2). The distributions of each item varied (following visual inspection of histograms for each item), some were normally distributed (feeling sleepy, fatigue, pain), whereas others were positively skewed (shortness of breath, problems using arms, dribbling saliva, amongst others). There was evidence for floor effects for all items except poor mobility, fatigue and problems using legs. Ceiling effects were seen only for poor mobility and problems using legs. The Cronbach’s alpha for the sample was 0.77, indicating good internal consistency.

**Table 2:** Frequency distribution of symptom scores.

Symptom	Mean (SD)	0 (*not at all*) % (n)	1 (*slightly*) % (n)	2 (*moderately*) % (n)	3 (*severely*) % (n)	4 (*over-whelmingly*) % (n)
Pain	1.6 (1.2)	23.7 (67)	21.2 (60)	30.4 (86)	21.6 (61)	3.2 (9)
Shortness of breath	0.8 (1.0)	50.5 (143)	25.1 (71)	18.7 (53)	4.2 (12)	1.4 (4)
Nausea	0.3 (0.7)	78.8 (223)	12.4 (35)	7.4 (21)	0.7 (2)	0.7 (2)
Vomiting	<0.1 (0.4)	97.2 (275)	1.4 (4)	0.7 (2)	—	0.7 (2)
Poor appetite	0.5 (1.0)	70.3 (199)	12.4 (35)	11.7 (33)	3.9 (11)	1.8 (5)
Constipation	1.2 (1.2)	37.1 (105)	19.1 (54)	28.3 (80)	13.1 (37)	2.5 (7)
Sore/dry mouth	1.0 (1.1)	47.0 (133)	17.0 (48)	25.1 (71)	8.5 (24)	2.5 (7)
Drowsiness	1.5 (1.1)	25.8 (73)	19.4 (55)	38.5 (109)	13.1 (37)	3.2 (9)
Poor mobility	2.8 (1.0)	3.9 (11)	6.7 (19)	22.3 (63)	41.7 (118)	25.4 (72)
Spasms	1.2 (1.2)	39.9 (113)	20.9 (59)	20.9 (59)	13.1 (37)	5.3 (15)
Fatigue	2.1 (1.1)	10.3 (29)	17.3 (49)	37.1 (105)	26.2 (74)	9.2 (26)
Problems swallowing	0.7 (1.0)	60.1 (170)	15.9 (45)	16.3 (46)	6.4 (18)	1.4 (4)
Feeling sleepy	1.8 (1.1)	16.6 (47)	20.5 (58)	35.0 (99)	21.6 (61)	6.4 (18)
Difficulty sleeping	1.1 (1.2)	48.4 (137)	14.1 (40)	22.6 (64)	12.0 (34)	2.8 (8)
Difficulty controlling bowels	0.9 (1.2)	54.1 (153)	21.2 (60)	12.4 (35)	7.1 (20)	5.3 (15)
Difficulty controlling urine	1.4 (1.3)	37.5 (106)	20.5 (58)	19.4 (55)	14.1 (40)	8.5 (24)
Pressure sores	0.3 (0.7)	84.5 (239)	7.0 (20)	5.3 (15)	2.5 (7)	0.7 (2)
Problems using arms	1.4 (1.2)	32.5 (92)	21.9 (62)	23.3 (66)	17.0 (48)	5.3 (15)
Problems using legs	2.6 (1.2)	8.8 (25)	9.2 (26)	19.1 (54)	39.6 (112)	23.3 (66)
Difficulty communicating	1.1 (1.1)	42.4 (120)	23.0 (65)	21.6 (61)	11.3 (32)	1.8 (5)
Dribbling saliva	0.9 (1.1)	49.8 (141)	23.7 (67)	13.8 (39)	10.3 (29)	2.5 (7)
Falls	0.8 (1.1)	60.4 (171)	16.6 (47)	13.1 (37)	7.1 (20)	2.8 (8)
Hallucinations	0.4 (0.8)	75.6 (214)	13.1 (37)	7.4 (21)	2.8 (8)	1.1 (3)
Mouth problems	0.6 (1.0)	69.3 (196)	13.4 (38)	10.3 (29)	5.3 (15)	1.8 (5)

### Differential Item Functioning

Symptom scores were compared in MS and IPD patients (as the two biggest patient groups in this sample) using differential item functioning (DIF) analysis (results presented in Supplementary Material). Using a sub-sample of 196 MS (N = 119) and IPD (N = 113) patients, DIF was found for nine of the 24 items: patients with MS were more likely to report poor appetite, spasms, difficultly controlling bowels, problems using arms and problems using legs and patients with IPD were more likely to report difficulty communicating, dribbling saliva, hallucinations and mouth problems.

### Factor analysis

The EFA retained eight factors after specifying a minimum eigen value of 1.0. However, less than 3 items loaded onto factors 5, 6, 7 and 8 (with factor loadings > 0.3), thus four factors were retained in the CFA. Falls was the only item to not load onto a factor and was excluded from the model.

Covarying error terms were added for the following items: drowsiness and feeling sleepy, poor mobility and problems using legs, sore/dry mouth and difficultly communicating, sore/dry mouth and dribbling saliva, sore/dry mouth and mouth problems, nausea and vomiting. The final model is shown in Table 3.

**Table 3:** Confirmatory factor analysis; Standardised factor loadings (Standard error).

	Factor One -Fatigue related symptoms	Factor Two -Motor related symptoms	Factor Three -Oral problems	Factor Four -Non-motor symptoms
Drowsiness	0.50 (0.06)			
Fatigue	0.84 (0.05)			
Feeling sleepy	0.63 (0.05)			
Difficulty controlling urine	0.30 (0.06)			
Constipation		0.30 (0.07)		
Poor mobility		0.59 (0.06)		
Spasms		0.50 (0.06)		
Difficulty controlling bowels		0.42 (0.06)		
Pressure sores		0.34 (0.07)		
Problems using arms		0.59 (0.06)		
Problems using legs		0.60 (0.06)		
Sore/dry mouth			0.61 (0.08)	
Problems swallowing			0.56 (0.06)	
Difficulty communicating			0.62 (0.06)	
Dribbling saliva			0.59 (0.06)	
Hallucinations			0.30 (0.06)	
Mouth problems			0.46 (0.06)	
Pain				0.51 (0.06)
Shortness of breath				0.33 (0.07)
Nausea				0.51 (0.06)
Poor appetite				0.48 (0.06)
Vomiting				0.37 (0.07)
Difficulty sleeping				0.44 (0.06)
Falls				
Fit statistics^a^	AIC = 17845.017, RMSEA = 0.045, CFI = 0.894, TLI = 0.876, SRMR = 0.064			

^a^AIC – Akaike information criterion.

RMSEA – Root mean square error of approximation.

CFI – Confirmatory fit index.

TLI – Tucker-Lewis Index.

SRMR – Standardised root mean squared residual.

The CFA produced a fairly good-fitting model, as indicated by the goodness of fit statistics (RMSEA = 0.045 and SRMR = 0.064 both indicated a good fit, the CFI (0.894) and TLI (0.876) are approaching adequate fit). Moreover, this model was robust when repeated for MS and IPD patients independently (see Supplementary Material).

Factors were mostly weakly to moderately correlated. Fatigue related symptoms were moderately correlated with motor related symptoms (0.45) and strongly correlated with non-motor symptoms (0.62). Non-motor symptoms were moderately correlated with motor related symptoms (0.44) and oral problems (0.40). Oral problems were weakly correlated with fatigue related symptoms (0.23) and motor related symptoms (0.06).

## Discussion

This is the first assessment of a range of symptoms across LTNCs using factor analysis, utilising baseline data from a multicentre clinical trial. The IPOS Neuro-S24 is a promising measure of symptoms across LTNCs and data using this measure produced a clinically meaningful four-factor model illustrating symptom dimensions related to fatigue, motor symptoms, oral problems and non-motor symptoms.

The first factor predominantly included fatigue related symptoms. The factor loadings were generally high for these symptoms. Similar results were reported in a cluster analysis of symptoms in cancer patients^17^, as fatigue, lack of energy and weakness clustered together. Although the statistical techniques differed, it could be concluded that across these patient groups, these symptoms tend to appear together.

The second factor represented symptoms relating to motor control and mobility. Although difficulty controlling bowels appears to be less similar to the other symptoms that load on to this factor, bowel problems may be a symptom of LTNCs due to nerve damage or loss of muscle control. Similarly, constipation can result from lack of physical activity. Problems relating to mobility frequently appear in the literature exploring symptomatology in LTNCs. In a systematic review of LTNC symptoms^4^, in 40 articles there were 18 different descriptors of mobility symptoms/problems and these symptoms were highly prevalent (>80% patients). The present study adds to this as the results demonstrate that symptoms presenting as the adverse effects of poor mobility (eg, pressure sores, constipation) load onto the same factor.

The third factor predominantly represented oral problems. Oral symptoms (including communication, swallowing and dribbling/drooling) were presented by Saleem *et al*.^4^ as commonly reported symptoms in the LTNC literature. The present study adds to this by suggesting that these symptoms often co-occur.

The last factor included non-motor symptoms. It also included gastro-intestinal symptoms. These results are supported by Fink *et al*.^18^, who found a gastro-intestinal factor in their analysis of symptoms in patients from a neurology clinic, general medicine and primary care.

Although most symptoms that load onto factors fit within a logical ‘theme’, there were seemingly miscellaneous symptoms, particularly difficulty controlling urine and hallucinations. Difficulty controlling urine loaded onto fatigue related symptoms, however this could be related to nocturia, muscle weakness and/or toileting problems due to poor mobility or lack of energy. Hallucinations, a neuropsychiatric non-motor symptom typically seen in IPD patients^19^, loaded onto oral problems. Swallowing dysfunction in IPD relates to more widespread Lewy bodies, or dry mouth due to anti-cholinergics may be a risk factor for confusion. Additionally, it is worth noting that many symptoms reported by patients may be related to treatments rather than the condition itself^3^. Thus, although factor analysis demonstrates where symptoms load onto common factors, it does not account for when one symptom might be caused by another, or may be symptomatic of a treatment for another symptom.

A key finding was the robustness of the factor model (and symptom dimensions) across different LTNCs. DIF was found for nine of the 24 items. However, it is worth noting that the Mantel-Haenszel tests used produced wide confidence intervals for spasms, problems using legs and hallucinations and mouth problems was borderline significant, indicating weak evidence for DIF. The CFA model fit the data (although to slightly less a degree) for MS and IPD patients independently. The models were comparable, however the fit statistics indicated the model fit the data best when using the whole sample, suggesting that the model was appropriate for use across LTNCs.

As this factor analysis was the first of its kind using a large number of motor and non-motor symptoms in this patient group, it is difficult to compare with other established measures. A factor analysis including patients with LTNCs using eight of the symptoms also used in the present study (pain, shortness of breath, nausea, vomiting, constipation, spasms, difficulty sleeping and mouth problems) reported a similar Cronbach’s alpha (0.66), also indicating good internal consistency^11^. As this scale comprised fewer items than the IPOS Neuro S-24, it presented fewer factors (two), which were not able to represent multiple symptom groups. It did, however, present good fit indices (SRMR = 0.057, RMSEA = 0.046), comparable to those seen in the present study. Two symptom measures identified in the literature^17,18^, each comprising 25 symptoms, though not using LTNC samples, demonstrated how symptoms were likely to co-occur in different patient groups. In a factor analysis of symptoms in both neurological and general medical patients, three factors were observed: cardiopulmonary, musculoskeletal and gastrointestinal symptoms. In a cluster analysis of symptoms in patients with cancer, seven symptom clusters were identified. These findings suggest that including a wider range of symptoms in a measure has the benefit of demonstrating if and which symptoms are likely to co-occur by indicating a number of symptom groups.

These results have implications for care and research in LTNCs. This study demonstrates that symptom dimensions are similar across LTNCs and that a single tool can be used to measure the outcome in this patient group. Moreover, the results may benefit clinical practice as symptom factors indicate that, where an individual reports experiencing one symptom, they are more likely to experience other symptoms that load on to the same factor. Better understanding of symptoms in LTNCs is necessary for the care of patients^3–5^, particularly palliative care, which is focused on symptom management and comfort. Previous research has found that collaborative working between neurology and palliative care professionals has room for improvement^20^; a palliative outcome measure specifically for patients with LTNCs could influence these working practices and patient outcomes in future.

This study benefitted from a relatively large clinical sample (N = 283) given that recruitment of palliative patients is difficult and numbers of LTNC patients are smaller compared to other incurable conditions. Potential limitations of the present study include exclusion of patients lacking capacity, as only patient-reported ratings were included in the factor analysis, and exclusion of patients with missing data. The IPOS Neuro-S24 comprises predominantly physical symptoms, rather than psychiatric, behavioural or cognitive symptoms. However, this was the nature of the measure, thus exploration of psychiatric, behavioural or cognitive symptoms was beyond the scope of this study.

The results from this study produced a reliable four-factor CFA model, using baseline data from a clinical sample of LTNC patients. Four stable symptom domains were identified: fatigue related symptoms, motor/mobility related symptoms, oral problems and gastric/other symptoms. Although floor effects were present, the IPOS Neuro S-24 showed promising psychometric properties and is an appropriate tool to measure symptoms in this patient group. This will enable health care providers to assess and monitor symptomatology in this patient group and provide appropriate and integrated neurological and palliative care.

## Supplementary information

Supplementary material

## Data Availability

The datasets generated during and/or analysed during the current study are not publicly available due to being sourced from an ongoing, yet unpublished trial, but are available from the corresponding author on reasonable request.
