# Supplementary material for: Symptom dimensions in people affected by long-term neurological conditions: a factor analysis of a patient-centred palliative care outcome symptom scale
Source: Sci Rep. 2019 Mar 21;9:4972. doi: 10.1038/s41598-019-41370-3 (PMC6428819; doi:10.1038/s41598-019-41370-3)
Supplement: Supplementary file 1 — Supplementary material [file 41598_2019_41370_MOESM1_ESM.pdf]

Symptom dimensions in people affected by long-term neurological conditions: a factor analysis of a patient-centred palliative care outcome symptom scale

Rebecca Wilson[1]*, Nilay Hepgul[1], Romi A Saha[2], Irene J Higginson[1] & Wei Gao[1]

[1] Cicely Saunders Institute of Palliative Care, Policy & Rehabilitation, Florence Nightingale Faculty of Nursing, Midwifery & Palliative Care, King's College London, Bessemer Road, London, SE5 9PJ, UK.

[2] Hurstwood Park Neurological Centre, Brighton and Sussex University Hospitals, Lewes Road, Haywards Heath, RH16 4EX, UK.

Supplementary material

Table S1 – Differential Item functioning (DIF) - Mantel-Haenszel DIF analysis, comparing symptom severity MS vs IPD patients

**Symptom**	**OR (95% CI)**
Pain	1.5 (0.7-3.2)
Shortness of breath	0.9 (0.4-1.6)
Nausea	1.0 (0.5-2.1)
Vomiting	1.8 (0.4-7.8)
Poor appetite	2.2 (1.1-4.5)*
Constipation	1.1 (0.6-1.9)
Sore/dry mouth	0.7 (0.4-1.3)
Drowsiness	0.8 (0.3-1.8)
Poor mobility	3.1 (0.4-22.7)
Spasms	5.9 (2.8-12.3)*
Fatigue	1.2 (0.4-3.9)
Problems swallowing	1.5 (0.7-3.0)
Feeling sleepy	0.8 (0.4-1.9)
Difficulty sleeping	1.1 (0.6-2.0)
Difficulty controlling bowels	2.9 (1.6-5.5)*
Difficulty controlling urine	0.7 (0.4-1.3)
Pressure sores	2.1 (0.9-5.0)
Problems using arms	2.6 (1.3-5.2)*
Problems using legs	6.4 (1.5-26.3)*
Difficulty communicating	0.4 (0.2-0.8)*
Dribbling saliva	0.2 (0.1-0.3)*
Falls	0.9 (0.5-1.6)
Hallucinations	<0.1 (<0.1-0.2)*
Mouth problems	0.5 (0.3-1.0)*

*p<.05

Table S2 – Confirmatory factor analysis (CFA) for MS patients (N=119)

	Factor 1	Factor 2	Factor 3	Factor 4
	Standardised factor loadings (Standard error)			
Drowsiness	0.57 (0.08)			
Fatigue	0.95 (0.08)			
Feeling sleepy	0.68 (0.07)			
Difficulty controlling urine	0.29 (0.09)			
Constipation		0.43 (0.10)		
Poor mobility		0.46 (0.10)		
Spasms		0.45 (0.11)		
Difficulty controlling bowels		0.39 (0.11)		
Pressure sores		0.40 (0.10)		
Problems using arms		0.57 (0.10)		
Problems using legs		0.37 (0.11)		
Sore/dry mouth			0.70 (0.10)	
Problems swallowing			0.56 (0.09)	
Difficulty communicating			0.55 (0.10)	
Dribbling saliva			0.50 (0.10)	
Hallucinations			0.32 (0.10)	
Mouth problems			0.20 (0.11)	
Pain				0.60 (0.09)
Shortness of breath				0.37 (0.10)
Nausea				0.63 (0.08)
Poor appetite				0.48 (0.09)
Vomiting				0.39 (0.10)
Difficulty sleeping				0.41 (0.10)
Falls				
Fit statistics	AIC = 7362.188<br>RMSEA = 0.040<br>CFI = 0.911<br>TLI = 0.896<br>SRMR = 0.078			

Table S3 – Confirmatory factor analysis (CFA) for IPD patients (N=113)

	Factor 1	Factor 2	Factor 3	Factor 4
	Standardised factor loadings (Standard error)			
Drowsiness	0.38 (0.11)			
Fatigue	0.73 (0.11)			
Feeling sleepy	0.52 (0.10)			
Difficulty controlling urine	0.23 (0.12)			
Constipation		0.29 (0.11)		
Poor mobility		0.62 (0.10)		
Spasms		0.28 (0.11)		
Difficulty controlling bowels		0.36 (0.11)		
Pressure sores		0.24 (0.11)		
Problems using arms		0.60 (0.09)		
Problems using legs		0.57 (0.10)		
Sore/dry mouth			0.39 (0.12)	
Problems swallowing			0.72 (0.08)	
Difficulty communicating			0.55 (0.09)	
Dribbling saliva			0.36 (0.10)	
Hallucinations			0.24 (0.11)	
Mouth problems			0.55 (0.09)	
Pain				0.38 (0.11)
Shortness of breath				0.45 (0.10)
Nausea				0.45 (0.11)
Poor appetite				0.49 (0.10)
Vomiting				0.41 (0.11)
Difficulty sleeping				0.52 (0.10)
Falls				
Fit statistics	AIC = 6925.567<br>RMSEA = 0.069<br>CFI = 0.748<br>TLI = 0.708<br>SRMR = 0.094			
